# The influence of functional electrical stimulation on hand motor recovery in stroke patients: a review

**DOI:** 10.1186/2040-7378-6-9

**Published:** 2014-08-21

**Authors:** Fanny Quandt, Friedhelm C Hummel

**Affiliations:** 1 Brain Imaging and Neurostimulation (BINS) Labor, Department of Neurology, University Medical Center Hamburg-Eppendorf, Martinistrasse 52, Hamburg W34 20246, Germany; 2 Favaloro University Av, Belgrano 1746, Buenos Aires C1093AAS, Argentina

**Keywords:** Functional electrical stimulation, FES, Neuromuscular electrical stimulation, NMES, Stroke, Rehabilitation, Upper extremity, Hand, Neuroprothesis

## Abstract

Neuromuscular stimulation has been used as one potential rehabilitative treatment option to restore motor function and improve recovery in patients with paresis. Especially stroke patients who often regain only limited hand function would greatly benefit from a therapy that enhances recovery and restores movement. Multiple studies investigated the effect of functional electrical stimulation on hand paresis, the results however are inconsistent. Here we review the current literature on functional electrical stimulation on hand motor recovery in stroke patients. We discuss the impact of different parameters such as stage after stoke, degree of impairment, spasticity and treatment protocols on the functional outcome. Importantly, we outline the results from recent studies investigating the cortical effects elicited by functional electrical stimulation giving insights into the underlying mechanisms responsible for long-term treatment effects. Bringing together the findings from present research it becomes clear that both, treatment outcomes as well as the neurophysiologic mechanisms causing functional recovery, vary depending on patient characteristics. In order to develop unified treatment guidelines it is essential to conduct homogenous studies assessing the impact of different parameters on rehabilitative success.

## Introduction

Neuromuscular electrical stimulation in patients with motor function impairment of the upper extremity has been employed as one rehabilitative treatment option for many years [1]. Electrical stimulation of the neuromuscular system induces a depolarization of peripheral neurons and subsequently elicits muscle contractions. One can distinguish between electrical stimulation used as a therapeutic intervention and functional electrical stimulation (FES). The former induces physiological changes that remain after the actual stimulation, facilitating plastic changes during recovery and leading to improvement of voluntary functions. In contrast, the primary goal of neuromuscular FES is to supplement lost functions. In FES muscles are stimulated in a coordinated manner with the objective to provide function. One of the best known applications is the peroneal nerve stimulator in patients with a drop foot [2]. Thus, FES systems are used to assist patients either by substituting or supporting movements. FES is often applied in patients whose functional recovery has already plateaued. However, it has been shown that repeated muscle activation within the framework of FES might also lead to improvement of voluntary motor control, which exceeded the time of stimulation, compounding the terms therapeutic and functional electrical stimulation and raising the question by what means FES influences motor recovery.

Most applications of functional electrical stimulation in the upper extremity have been targeted towards patients with spinal cord injury (SCI) [3-5]. In SCI patients it is the main goal to substitute lost hand functions, in particular the ability to grasp, hold and release objects [3,6]. Since in stroke patients weakness of distal muscles is usually severe and lacks sufficient recovery [7] electrical stimulation became of interest as one treatment option for stroke patients with significant motor deficits of the upper extremity. Even though, numerous studies found a positive impact of neuromuscular electrical stimulation for hand motor recovery after stroke [8-15], strong evidence underpinning the efficiency of neuromuscular electrical stimulation is still missing. A recent Cochrane review reported the superiority of electrical stimulation compared to no treatment, however, could not identify an advantage over other treatment options such as conventional physical therapy [16]. Nonetheless, electrical stimulation should not be abandoned as one potential treatment option in stroke rehabilitation, especially since studies show great heterogeneity complicating an overall conclusion.

This review provides an overview of the rehabilitative effect of functional electrical stimulation on hand and finger function in stroke patients. We focus on the current opinion of how electrical stimulation influences motor recovery and in particular its effect on the central reorganization of movement patterns. Furthermore, we give insight into the differences between studies, namely means of control, patients groups, treatment protocols and stimulation parameters and discuss their impact on long-term treatment success.

## Review

### Technical implementation of functional electrical stimulation

#### ***Configuration of a FES-based neuroprothesis***

The configuration of FES-based neuroprosthetic systems can be distinguished regarding the number and location of stimulation electrodes. Most studies employ surface electrodes placed on the skin (transcutaneous systems), nonetheless, there are also percutaneous [17] as well as fully implantable systems [18]. Surface electrodes are non-invasive, easily applied and reversible, however, stimulation of specific muscles is relatively unselective and hand movement can lead to displacement of the muscles relatively to the electrodes. Implantable systems allow greater selectivity and stimulation of deeper muscles, yet application of invasive electrodes is limited because of the need for surgery and the risk of infection. In transcutaneous systems, the active surface electrode is placed over the muscle motor point that evokes the desired movement with the least current necessary. In a monopolar set-up the indifferent electrode is placed over less excitable tissue, for example a tendon or fascia. Here, multiple active electrodes can be referenced to one indifferent electrode. In a bipolar set-up two electrodes are placed in closer proximity providing the advantage of a more localized electric field, per contra each active electrode requires its own reference electrode. Recent attempts to optimize stimulation for effective grasping led to the development of multi-pad electrode systems which facilitate more selective and localized stimulation [19-21]. Future research should further optimize multi-pad systems towards an individual adjustment regarding electrode size, shape, position and stimulation pattern. Moreover, efforts are made to incorporate electrodes into a predefined fabric sleeve reducing the amount of time needed to install the electrode set-up [22]. Another approach to enhance the functionality of stimulation is by combining the FES-based neuroprothesic system with an orthosis providing stability and guidance of the joints [23,24]. One commercially available upper-extremity neuroprothesis is the H200 Wireless Hand Rehabilitation System by Bioness which incorporates electrical stimulation with a wrist and hand orthosis (as used in [15,25,26]). Other available systems, however without an additional orthosis, include Automove AM800 (Krauth + Timmermann, GER), NeuroMove NN900 (Biomation, USA) and MyoTrac Infiniti (Saebo, USA).

#### ***Stimulation parameters***

The specific magnitude parameters of stimulation are subject of discussion. There is scope for variation not only across studies but also inter-individually to evoke optimal muscle contraction and avoid discomfort, pain and skin irritations. The waveform of electrical current pulses is defined by the amplitude (mA), pulse width (μs), ramping form and frequency (Hz). Frequencies above 12 Hz – 15 Hz lead to a temporal summation of muscle twitches, increasing the strength and smoothness of a muscle contraction. Spatial summation is achieved by increasing the electrical charge through pulse amplitude and pulse width, influencing the depth of the stimulation effect. Often frequency and pulse width are set constant, whereas pulse amplitude is varied. Stimulation parameters for successful FES are in the range of (I) frequency: 20 – 50 Hz, (II) pulse width: 30 – 500 μs, (III) amplitude ≤ 100 mA [14,27,28]. De Kroon et al. compared the influence of different stimulation frequencies across studies and detected no relationship between frequency and clinical outcome [27]. A more recent study investigated the effect of pulse width on the paretic and non-paretic arm and found a significant increase in elbow flexion torque in the paretic arm when using a wide-pulse stimulation scheme (1000 μs) [29]. The authors speculate that wide-pulse stimulation may lead to a greater reflexive recruitment in the paretic arm, underlining that pathological changes influence stimulation response.

One limitation is the rapid development of muscle fatigue. During electrical stimulation multiple motor unites are stimulated synchronously in a non-selective order whereas during natural behavior there is an unsynchronized de- and recruitment of motor units with an initial recruitment of smaller fatigue-resistant units. This pattern of motor recruitment in electrically evoked contractions provokes muscle fatigue, which is enhanced with high stimulation frequencies. One improvement are the above mentioned multi-pad arrays which enable asynchronous stimulation leading to a superposition of muscle force but not action potentials, reducing emerging muscle fatigue [19].

### The impact of electrical stimulation on motor recovery and its implication for FES-based rehabilitation

#### ***Long-term changes after FES and changes in cortical activation patterns***

Since some patients exhibited benefits exceeding the time of stimulation, research has emerged investigating the effects of FES on cortical activation patterns [9,30-34]. While all studies found changes of neural activation after repetitive FES use, study designs and results are inhomogeneous. In general, there are two trends of results across studies. On the one hand, some studies found either an increase of fMRI BOLD signal in the contralesional postcentral gyrus [9,34] as a correlate of enhanced cortical activation or a decrease of activation in the ipsilesional hemisphere correlated to reduced cortical activation [31]. On the other hand, other research groups propose that FES, accompanied by improvement of motor function, leads to less diffuse activity in the sensorimotor cortex (SMC), shifting to focused activity in the ipsilesional SMC [32,33]. Likewise, a recent study investigating changes of cortical brain perfusion using near-infrared spectroscopy (NIRS) during and after therapeutic FES intervention found a redistribution towards the ipsilesional SMC in patients with improved functional motor control following FES [30]. Differences in findings could be attributed to heterogeneous patient groups with regard to time after stroke as well as stroke location, degree of impairment and integrity of the corticospinal tract [35]. Reviewing the studies assessing cortical effects upon FES, one can observe a trend towards severe impairment leading to activation of the contralesional site, whereas less impaired patients tend to recruit the ipsilesional site (Table 1). This is in line with the current literature of cortical reorganization during stroke recovery, describing the relationship of lateralization and functional outcome [36-38]. Good recovery of hand function is correlated with an increase of ipsilesional brain activity over time, whereas larger strokes with worse outcomes engage contralesional sites.

**Table 1:** Overview over studies assessing cortical effects of FES

	**Post stroke**	**Degree of impairment**	**Method**	**Outcome**
Hara et al. 2013 [30]	> 1 year	FM: 24 (11 – 37); > 20° extension of the 3^rd^ finger	NIRS	ipsilesional SMC ↑
Shin et al. 2008* [33]	> 1 year	BBT: 21.14 ± 4.09; > 20° extension of the 3^rd^ finger	fMRI	contralesional SMC ↓
Sasaki et al. 2012 [32]	> 1 year	FM: 41.8 ± 5.08	fMRI	contralesional SMC ↓ (no statistics)
Page et al. 2010 [34]	> 6 month	FM: 23 (6 – 35); no active extension of fingers or wrist	fMRI	contralesional SMC ↑
Kimberley et al. 2004* [9]	> 6 month	> 10° extension of the 2^nd^ finger	fMRI	contralesional SMC ↑
Wei et al. 2013 [31]	2 – 6 weeks	FM: 30 (6-50); no active extension of fingers	fMRI	ipsilesional SMC ↓

Comparison of studies assessing the cortical effects of FES treatment in relationship to post stroke time point, degree of impairment and methods of evaluation. One can observe a trend towards severe impairment leading to activation of the contralesional site, whereas less impaired patients tend to recruit the ipsilesional site. Studies marked with an asterisk (*) are randomized controlled trials. FM = Fugl-Meyer Assessment of the upper extremity; BBT = Box and Block-Test; NIRS = Near-infrared spectroscopy; fMRI = functional magnetic resonance imaging; SMC = sensorimotor cortex.

#### ***Mechanisms by which FES could lead to plastic changes***

It remains the question, however, by what means FES influences cortical reorganization. Multiple mechanisms have been proposed. Firstly, FES not only stimulates motor nerve fibers but also afferent sensory nerve fibers. Alteration of afferent input has been shown to lead to organizational changes in cortex in rodents [39] and prolonged stimulation of peripheral nerves can induce changes of motor networks in human cortex [40]. Therefore, the afferent feedback provided by FES temporally coupled with task-related motor execution could facilitate brain plasticity. Secondly, electrical stimulation also induces antidromic firing of motor nerve fibers. The retrograde impulse leads to a depolarization of the anterior horn cells. Rushton hypothesized that the synapse of the pyramidal tract to the anterior horn cell may act as a modifiable Hebb-type synapse in which the antidromic discharge triggered by FES coupled with voluntary motor effort lead to a synchronized pre- and postsynaptic coupling and enhanced synaptic remodeling [41]. Regardless the precise mechanism underlying reorganization, neural network changes during recovery after injury seem to be similar to changes during motor learning of a new skill [42-44]. Cortical reorganization during motor learning is evoked by repeatedly performed skilled movements with task specificity and high functional content [45,46]. Moreover, the visual-perception information of the electrically evoked movement can further enhance motor learning [47]. Consequently, functional electrical stimulation could enhance motor recovery on the grounds that it simulates a meaningful task and increases functional relevance.

#### ***EMG-mediated FES***

The onset of stimulation can be triggered either cyclic, by button press or through movement-related EMG activity exceeding a predefined threshold. EMG-mediated FES has been applied in multiple studies [12,30,48-50] and links voluntary movement attempts to electrical stimulation. Studies suggested that EMG-mediated functional stimulation is more likely to improve motor functions than cyclic stimulation [13]. One can speculate that temporally coupling central neural activity, here the intention to execute a movement, with peripheral activity, a stimulated motor output, reinforces plastic changes further compared to sole stimulation [51,52]. Since stroke patients with severe paresis might fail to evoke sufficient myographic activity, Hong et al. combined EMG-triggered FES with mental imagery training to heighten the electrical activity produced by the muscle and observed an advantage over FES alone [53]. In the attempt to enhance neuroplasticity, other research groups decoded cortical changes during motor imagery using EEG to drive electrical stimulation [54,55].

#### ***Stage after stroke***

It remains an open research question to what degree the delay of FES intervention after stroke is critical for rehabilitative success. The time course of cortical reorganization following stroke has been studied in animals and humans (for a review see [56]). During the early post-stroke recovery phase multiple mechanisms, such as homeostatic processes, altered gen expression and changed overall excitability have been shown to promote neuroplasticity [57,58]. The post-stroke brain displays enhanced sensitivity to rehabilitative treatment, hence making the time point for treatment initiation critical [59]. Even during the chronic stage, however, recovery of motor function can be obtained by enhanced rehabilitative therapy [60,61]. Since the primary goal of FES rehabilitation is directed towards the supplementation of lost function, most studies investigated the effect of FES in chronic stroke patients [9,28,30,33,62-64]. Only few studies looked at the rehabilitation effect of FES in acute stroke patients during a time period with potentially even greater impact on recovery (Table 2, [10,14,25,65-67]). A randomized controlled study by Malhotra et al. investigated the effects of neuromuscular electrical stimulation in acute stroke patients with no functional arm movements and found that treatment prevented the development of pain in patients and resulted in improvement of muscle strength [14,66]. To make a comparison across results more complex, especially in studies probing the recovery in a heterogeneous group of acute patients, it is difficult to distinguish between spontaneous improvement or improvement due to a given intervention. All in all, it remains unclear if and how the stage after stroke impacts the potential for improvement mediated through FES. As described earlier, the changes in cortical activation patterns and hence the rehabilitative mechanisms might be dependent on the stage after stroke. So far no studies have been conducted specifically comparing the differences in acute, subacute and chronic stroke patients.

**Table 2:** Treatment protocols of neuromuscular rehabilitation in acute stroke patients

	**Time of onset**	**Frequency per week**	**Duration per session**	**Duration of intervention**
Malhotra et al. 2013* [14]	< 6 weeks	5	2 × 30 min	6 weeks
Mangold et al. 2009* [67]	2 – 18 weeks	3	45 min	4 weeks
Alon et al. 2007* [25]	< 30 days	5	4 × 10 min increase of 5 min per day	12 weeks
Chae et al. 1998* [65]	< 22 days	7	60 min	15 sessions
Francisco et al. 1998* [10]	< 6 weeks	5	2 × 30 min	5 weeks

Summary of treatment protocols employed in FES studies evaluating acute stroke patients. Frequency of stimulation, duration per session and overall duration of intervention vary greatly across studies. Studies marked with an asterisk (*) are randomized controlled trials.

#### ***Degree of impairment***

Most studies utilizing FES combine a great variety of patients with diverse severity ranging from mild impairments of dexterity to no residual hand motor function. Thus, comparison of FES studies is complicated by heterogeneous inclusion criteria, which is reflected in diverse outcome results [68]. Von Lewinski et al. found a trend towards greater improvement of arm function in chronic stroke patients with only mild to moderate motor impairment [50]. A clear conclusion, however, whether mild, moderate or severe impairment levels show a greater outcome effect cannot be drawn since most controlled, randomized-trials include only patients with mild to moderate impairment of hand function [8-11,64,65,69,70], whereas severely impaired patients, with no active hand extension, are predominately evaluated in single case and non-controlled studies [54,63,71]. Hence, FES-based rehabilitation would benefit from measures allowing an a priori prediction of responder-rate and functional recovery to help guide specific treatment protocols. However, the temporal evolution of motor function recovery as well as long-term outcome show large inter-individual differences. One simple indicator of functional outcome prognosis is the degree of initial motor impairment [72], however, individual prediction remains difficult due to notable variability in each patient. Newer measures such as the integrity of the corticomotor pathway and ipsilesional cortical activity after stroke obtained with neuroimaging techniques and TMS give rise to additional prognostic values [35,73,74], but it remains to be seen whether they can contribute information on treatment success using FES.

#### ***Spasticity***

Other parameters affecting usability of FES in stroke patients are spasticity and concurring muscle changes. On the one hand studies employing FES aim for a reduction of spasticity, on the other hand, excessive spasticity might complicate successful electrical stimulation and is an exclusion criteria in some studies [26,28,30,33]. Poststroke spasticity is a common complication occurring in roughly one third of stroke patients [75]. The pathophysiology of spasticity is likely due to abnormalities on different levels, including muscle properties, spinal mechanism as well as supraspinal alterations [76]. It is one hypothesis that electrical stimulation induces specific plasticity of spinal chord pathways [77] and first attempts to measure changes in spinal circuits through differences in the H-reflex upon neuromuscular electrical stimulation have been made [78]. However, even though few studies indicated a decrease in spasticity after treatment with FES [15,79], others did not find a reduction of spasticity compared to a control group [14,18]. Another obstacle for successful stimulation are chronic tissue changes due to immobilization such as atrophy, loss of sarcomeres, muscle conversion to connective tissue and a decreased resting length of the muscle [80]. Moreover, a loss of motor units in the paretic arm, which might be due to secondary trans-synaptic degeneration, could compromise effective FES performance [81,82]. In addition, concurrent flexor spasticity might reduce the effectiveness of FES and enhance spasticity further. Especially EMG-controlled FES with the patients trying to assist the stimulation with a voluntary movement effort [18,83] resulted in less effective stimulation effects due to flexor spasticity.

#### ***Dosages***

Not only the onset of treatment but also the duration and frequency of treatment are likely to influence motor outcome. Treatment protocols vary from only few days [9] up to multiple months [30] and daily duration of stimulation differs along studies (see Table 2 for an overview of studies in acute stroke patients).

Page et al. compared the efficacy of different rehabilitation protocols in subacute stroke patients and found that 120 minutes of repetitive task-specific practice combined with electrical stimulation was superior compared to shorter durations of stimulation [26]. Similarly, Hsu et al. reported the dosage, ranging from 0 – 20 hours of stimulation, as one positive determinant for functional improvement [84]. However, standardized protocols specifying frequency and duration of treatment are missing. One advantage of FES-based rehabilitative therapy is its possibility for in-home usage, allowing for higher cost-effective training intensities [15,48].

## Conclusions

Here, we reviewed the literature on functional electrical stimulation as one potential treatment option to improve motor recovery after stroke. Firstly, we summarized various configuration options of FES-based neuroprotheses, secondly we provided insight into the current view how FES influences motor recovery after stroke and lastly discussed various factors that might influence functional outcome and rehabilitative success.

Study results looking at the cortical effect after repetitive FES use are diverse, however, there is a trend pointing towards a lateralization of activity that is dependent on severity of impairment. Patients retaining some finger extension tend to shift towards focused activity in the ipsilesional site after FES, whereas patients who did not regain finger extension showed enhanced involvement of the contralesional site. The precise mechanisms contributing to neuronal plasticity remain vague. It is hypothesized that either concurrent stimulation of afferent fibers, or antidromic stimulation lead to enhanced synaptic remodeling, concrete evidence, however, is still lacking. Moreover, the precise timing of stimulation might be crucial for cortical changes, which is supported by findings demonstrating the advantage of EMG-triggered FES over cycling FES.

Even though studies indicate a potential benefit of FES-based therapy compared to either physical therapy or no therapy, strong evidence supporting an advantage of FES is still missing. As pointed out throughout the review, treatment protocols across studies are highly heterogeneous limiting the ability to generalize the results. No clear trend allowing a pre-selection of patients (stage after stroke, level of impairment) that would profit from FES could be detected. Regarding treatment protocols studies suggest an enhancement of recovery with prolonged FES use. There is need for determination of optimal stimulation parameters, which seem to be highly individual and also influenced by pathological changes of the neuromuscular transmission in the paretic hand.

All in all, future research needs to pinpoint the exact mechanisms underlying functional recovery after FES. Importantly, it is necessary to conduct homogenous randomized-controlled studies tackling the influence of impairment, timing of intervention and dosage. An a priori selection of suitable patients could help guide rehabilitation and optimization of individual treatment protocols to achieve larger treatment effects.

## Competing interests

The authors declare that they have no competing interests.

## Authors’ contributions

F.Q. carried out the literature review and drafted the manuscript. F.C.H helped to draft the manuscript and revised it critically for intellectual content. Both authors read and approved the final manuscript.
